# Cystine and Methionine Deficiency Promotes Ferroptosis by Inducing B-Cell Translocation Gene 1

**DOI:** 10.3390/antiox10101543

**Published:** 2021-09-28

**Authors:** Il-Je Cho, Doyeon Kim, Eun-Ok Kim, Kyung-Hwan Jegal, Jae-Kwang Kim, Sang-Mi Park, Rongjie Zhao, Sung-Hwan Ki, Sang-Chan Kim, Sae-Kwang Ku

**Affiliations:** 1 College of Korean Medicine, Daegu Haany University, Gyeongsan 38610, Korea; skek023@dhu.ac.kr (I.-J.C.); dy940716@gmail.com (D.K.); keo84@hanmail.net (E.-O.K.); outshinerz@gmail.com (K.-H.J.); kimjk@kiom.re.kr (J.-K.K.); miya38@nate.com (S.-M.P.); 2 Digital Health Research Division, Korea Institute of Oriental Medicine, Daejeon 34054, Korea; 3 Korean Medicine-Application Center, Korea Institute of Oriental Medicine, Daegu 41062, Korea; 4 Department of Psychopharmacology, Qiqihar Medical University, Qiqihar 161006, China; Zhao_rongjie@yahoo.com; 5 College of Pharmacy, Chosun University, Gwangju 61452, Korea; shki@chosun.ac.kr

**Keywords:** activating transcription factor 4 (ATF4), B-cell translocation gene 1 (BTG1), cystine and methionine deficiency (CST/Met (−)), ferroptosis, hepatocyte-derived cells

## Abstract

Ferroptosis is a type of programmed necrosis triggered by iron-dependent lipid peroxidation. We investigated the role of B-cell translocation gene 1 (BTG1) in cystine and methionine deficiency (CST/Met (−))-mediated cell death. CST/Met (−) depleted reduced and oxidized glutathione in hepatocyte-derived cells, increased prostaglandin-endoperoxide synthase 2 expression, and promoted reactive oxygen species accumulation and lipid peroxidation, as well as necrotic cell death. CST/Met (−)-mediated cell death and lipid peroxidation was specifically inhibited by pretreatment with ferroptosis inhibitors. In parallel with cell death, CST/Met (−) blocked global protein translation and increased the expression of genes associated with the integrated stress response. Moreover, CST/Met (−) significantly induced BTG1 expression. Using a BTG1 promoter-harboring reporter gene and siRNA, activating transcription factor 4 (ATF4) was identified as an essential transcription factor for CST/Met (−)-mediated BTG1 induction. Although knockout of BTG1 in human HAP1 cells did not affect the accumulation of reactive oxygen species induced by CST/Met (−), BTG1 knockout significantly decreased the induction of genes associated with the integrated stress response, and reduced lipid peroxidation and cell death in response to CST/Met (−). The results demonstrate that CST/Met (−) induces ferroptosis by activating ATF4-dependent BTG1 induction.

## 1. Introduction

Among the macromolecules in the cell, sulfur is a unique element found only in proteins. Methionine (Met) is an essential amino acid in mammals that must be obtained from external sources. By contrary, cysteine (Cys) can be synthesized from Met or obtained via degradation of imported cystine (CST; oxidized dimer of Cys). Although Cys is not essential, it can restore the glutathione content and reverse abnormal expression of albumin and drug-metabolizing enzymes in the hepatic tissue of experimental animals fed a low-protein diet [1,2,3]. In addition, Cys supplementation promotes human health by enhancing glucose clearance, reducing tumor necrosis factor α, and improving immune function [4].

Prolonged deficiency of sulfur amino acids attenuates protein synthesis and abolishes antioxidant capacity. Ferroptosis is a specific type of programmed necrosis provoked by iron-mediated lipid peroxidation [5,6]. Oxidative stress, inactivation of glutathione-dependent peroxide-scavenging enzymes (e.g., glutathione peroxidase 4), depletion of glutathione due to inhibition of substrates such as CST and sulfur amino acid deprivation, and suppression of glutathione-generating enzymes trigger ferroptosis [5,6,7,8,9,10,11,12]. In addition, branched-chain amino acid aminotransferase 2, MESH1, and ZIP7 have been recently identified as molecular determinants for modulating ferroptosis [13,14,15]. Because hepatocytes store large amounts of iron, dysregulated iron homeostasis increases their susceptibility to ferroptotic death. Thus, ferroptosis has been known to be involved in the pathogenesis of various liver diseases, such as acetaminophen-induced acute liver failure, ischemia/reperfusion injury, nonalcoholic steatohepatitis, fibrosis, and hepatocellular carcinoma [16]. However, novel determinants for regulating hepatic ferroptosis have been poorly understood.

The integrated stress response (ISR) is a conserved signaling pathway in mammals that mediates adaptation to diverse stresses such as nutrient deprivation, hypoxia, viral infection, oncogenic activation, and endoplasmic reticulum stress [17]. The ISR not only inhibits global protein translation via the phosphorylation of eukaryotic translation initiation factor 2α (eIF2α), but also activates the transcription of specific genes by cap-independent alternative translation of activating transcription factor 4 (ATF4) for adapting stressful condition [17,18,19]. Especially, ATF4 induction facilitates cell proliferation and acquires drug resistance via upregulating system X_c_^-^ antiporter [20,21]. However, ISR beyond adaptive capacity (e.g., duration and intensity) switches on ATF4-dependent signaling, triggering cell death. Although CST deficiency activates the ISR and simultaneously induces ferroptotic death [11,22], little attention has been paid to crosstalk between ISR and ferroptosis.

B-cell translocation gene 1 (BTG1), a member of the BTG/transducer of ErbB2 family initially identified as a juxtaposed gene of myc in B-cell chronic lymphocytic leukemia [23], is involved in regulating cell cycle progression, differentiation, and cell death by interacting with diverse transcriptional/translational modulators, such as ATF4, HoxB9, nuclear receptors, protein arginine methyltransferase 1 (PRMT1), and CCR4-associated factor 1 [24,25,26,27,28,29,30]. In addition, BTG1 is differentially induced in the liver of mice fed a low-protein diet [1], and its low expression is associated with a poor prognosis for hepatocellular carcinoma and nonalcoholic fatty liver disease [27,31]. By contrary, ectopic expression of BTG1 alleviates steatosis and improves insulin sensitivity in the liver [26,27]. Moreover, BTG1 induction is accompanied by deprivation of glucose and certain amino acids [1,25,27], suggesting that it is a novel molecular sensor involved in the response to nutrient deprivation. However, the signaling pathway associated with BTG1 induction is unclear.

Because HepG2 cells cannot synthesize Cys from Met while retaining the biochemical features of primary hepatocytes [32], the cells are useful for studying the role of sulfur amino acid deficiency in hepatocyte pathophysiology. The present study investigated the effect of CST and Met deficiency (CST/Met (−)) on ferroptotic death of hepatocyte-derived cells, primarily using HepG2 cells. In addition, we explored the signaling pathways involved in BTG1 induction, and the role of BTG1 under sulfur amino acid deficiency.

## 2. Materials and Methods

### 2.1. Reagents

BODIPY^®^ 581/591 undecanoic acid (C_11_-BODIPY) and an anti-BTG1 antibody (Cat. No., PA5-25035) were supplied by Thermo Fisher Scientific (Rockford, IL, USA). Z-VAD-FMK (Z-VAD; a pan-caspase inhibitor) and an anti-puromycin antibody (Cat. No., MABE343) were provided by Calbiochem (San Diego, CA, USA) and Merk Millipore (Billerica, MA, USA), respectively. An antibody for ATF4 was obtained from Proteintech (Cat. No., 60035-1; Chicago, IL, USA), and an antibody for phosphorylated general control non-derepressible 2 (GCN2; Thr^899^) was from Abcam (Cat. No., ab75836; Cambridge, UK). Primary antibodies for phosphorylated eIF2α (Ser^51^) (Cat. No., 9721) and eIF2α (Cat. No., 9722) and horseradish peroxidase-conjugated secondary antibodies (Cat. No., 7074 and 7076) were supplied by Cell Signaling Technology (Beverly, MA, USA). _L_-Cystine dihydrochloride, puromycin, control siRNA (si-control), and siRNAs targeting human and mouse ATF4 (si-ATF4) were provided by Santa Cruz Biotechnology (Santa Cruz, CA, USA). Thiazolyl blue tetrazolium bromide (MTT), _L_-methionine, anti-β-actin antibody (Cat. No., A5316), deferoxamine mesylate (DFX; an iron chelator), ferrostatin-1 (Fer-1; a ferroptosis inhibitor), necrostatin-1 (Nec-1; a necroptosis inhibitor), propidium iodide (PI), 2′,7′-dichlorofluorescein diacetate (DCFH-DA), ISR inhibitor (ISRIB), and other reagents were purchased from Sigma-Aldrich (St. Louis, MO, USA).

### 2.2. Cell Culture and Treatment

HepG2 and L-O2 cells (human hepatocyte-derived cell lines) were supplied by the American Type Culture Collection (Manassas, VA, USA) and China Cell Culture Center (Shanghai, China), respectively. Wild type (WT) HAP1 cells (a human haplotype cell line) were obtained from Horizon Discovery (Cambridge, UK). To generate BTG1 knockout (KO) cells, the gene was edited from WT HAP1 cells using gRNA (5′-TATGGTTGATGCGAATACAA-3′) and the CRISPR/Cas9 system (Horizon Discovery). A 1-bp deletion in exon 2 of the BTG1 gene was verified by DNA sequencing. According to the supplier’s instructions, all cells were maintained in an appropriate culture medium with 10% fetal bovine serum (HyClone Laboratories; Logan, UT, USA) and 1× antibiotic-antimycotic solution (Thermo Fisher Scientific). After cells in multiwell plates had been washed with phosphate-buffered saline, sulfur amino acids were depleted by incubating the cells with Dulbecco’s modified Eagle’s medium (DMEM) without CST and Met (Thermo Fisher Scientific) for the indicated time periods. Control cells were maintained in regular DMEM for the same periods. For some experiments, CST (200 μM), Met (200 μM), DFX (200 μM), Fer-1 (10 μM), Nec-1 (10 μM), Z-VAD (20 μM), or ISRIB (1 μM) were used in conjunction with DMEM without CST and Met.

### 2.3. Measurement of Glutathione

Levels of reduced glutathione (GSH) and oxidized glutathione (GSSG) were determined by GSH/GSSG-Glo^TM^ Assay (Promega, Madison, WI, USA), as described previously [33].

### 2.4. MTT Assay

The morphology of treated cells was observed using an Eclipse Ti-U microscope (Nikon; Kanagawa, Tokyo, Japan). Adherent cells were incubated with MTT (0.5 mg/mL) for 2 h, and formazan crystals were dissolved by adding dimethyl sulfoxide. The absorbance at 570 nm was measured using a Synergy HTX microplate reader (BioTek; Winooski, VT, USA), and relative cell viability was calculated as a percentage of the untreated controls.

### 2.5. Measurement of Necrotic Cell Death

Cells were stained with 1 μg/mL PI for 30 min, detached from the plate, collected by centrifugation, and resuspended in phosphate-buffered saline containing 1% fetal bovine serum. The proportion of 10,000 cells with high PI fluorescence intensity was analyzed using a flow cytometer (Partec, Münster, Germany). In addition, necrotic cell death was monitored using a Realtime-Glo^TM^ Apoptosis and Necrosis Assay (Promega). Briefly, cells were treated with CaCl_2_ and Necrosis Detection Reagent after replacing the medium without CST and Met. Fluorescence intensity was measured at 485 nm emission and 530 nm excitation wavelengths using a microplate reader (Infinite 200 Pro, Tecan; Männedorf, Switzerland).

### 2.6. Quantitative Polymerase Chain Reaction Analysis

Extraction of total RNA, cDNA synthesis using dT_16_, quantitative polymerase chain reaction (qPCR), and relative quantification of specific genes were performed as described previously [26]. The oligonucleotide sequences used for amplifying specific genes are listed in Table 1.

### 2.7. Measurement of Reactive Oxygen Species and Lipid Peroxidation

To determine reactive oxygen species (ROS) production, cells cultured in a 96-well black plate were incubated with 10 μM DCFH-DA for 1 h, and dichlorofluorescein fluorescence intensity was measured at 485 nm (emission)/530 nm (excitation) using a microplate reader (Tecan). Lipid peroxidation was measured after cells had been incubated in 3 μM C_11_-BODIPY for 1 h. The mean green fluorescence intensity of 10,000 cells was analyzed by an Accuri^TM^ C6 Plus flow cytometer (BD Biosciences; San Jose, CA, USA).

### 2.8. Immunoblot Analysis

Protein extraction using radioimmunoprecipitation buffer, sodium dodecyl sulfate-polyacrylamide gel electrophoresis, electrotransfer of proteins onto a nitrocellulose membrane, incubation of the membrane with appropriate antibodies, and chemiluminescence detection were conducted as described previously [33]. For surface sensing of translation (SUnSET) assay, HepG2 cells under CST/Met (−) were incubated with 1 μg/mL puromycin for 30 min, and puromycin-integrated polypeptides were detected using an anti-puromycin antibody. The expression of proteins was calculated relative to the band intensity of β-actin. Original blots are presented in Figure S3. 

### 2.9. Plasmid Construction, Transient Transfection, and Reporter Gene Assay

An expression plasmid encoding mouse ATF4 was a gift from Dr. David Ron (Addgene plasmid #21845). To generate luciferase reporter plasmids under the control of the BTG1 promoter, genomic DNA was isolated from Raw264.7 or HepG2 cells. According to Bakker and colleagues [34], the mouse BTG1 promoter region from −1015 to +96 bp (where −1 indicates the first upstream nucleotide of the transcriptional start site) was amplified and ligated into the XhoI and BglII sites of pGL-4.15[luc2P/Hygro] (Promega) for constructing mpBTG1-luc. Using a QuickChange Lightning Site-Directed Mutagenesis Kit (Agilent Technologies; Santa Clara, CA, USA), two putative ATF4 response elements in the mouse BTG1 promoter region were deleted from mpBTG1-luc to generate mpBTG1-ΔA4RE-luc. In addition, the human BTG1 promoter from −1190 to +56 bp was also inserted into pGL-4.15[luc2P/Hygro] to generate hpBTG1-luc. The primer pairs used for constructing the plasmids are listed in Table 1, and the DNA sequences of recombinant plasmids were verified using an ABI 3730 XL DNA sequencer. BTG1 reporter plasmid (300 ng), pRL-TK (a reporter gene driving constitutive expression of *Renilla* luciferase; 30 ng), and ATF4 (300 ng) were co-transfected into HepG2 cells for 24 h using FuGENE^®^ HD transfection reagent (Promega). Instead of the ATF4 expression plasmid, an equal amount of pCDNA3.2/V5-DEST (Thermo Fisher Scientific) was used for mock transfection. Luciferase activity was measured using a Dual-Luciferase^®^ Reporter Assay System (Promega). For some experiments, HepG2 cells were additionally transfected with si-control or si-ATF4 (100 pmol each) in the presence or absence of BTG1 reporter and ATF4 expression plasmids.

### 2.10. In Silico Analysis of BTG1 Promoter

After collecting the promoter sequences of the human, mouse, and rat BTG1 genes from −1.2 kb to −1 bp using the UCSC genome browser (https://genome.ucsc.edu (accessed on 7 June 2021)), putative sites capable of binding to human ATF4 (Matrix ID, MA0833.1 and MA0833.2) were scanned using JASPAR2020 (http://jaspar.genereg.net (accessed on 7 June 2021)) at a relative score threshold of 0.85.

### 2.11. Statistical Analysis

Numerical values are means ± standard deviation of at least three separated experiments. SPSS Statistics for Windows (Version 23.0, SPSS Inc.; Chicago, IL, USA) was used to compare means. Student’s *t*-test, analysis of variance, or Welch’s test was performed depending on the number of experimental groups and the homogeneity of variance. Tukey’s honestly significant difference or Dunnett’s T3 test was used for post hoc analysis. Statistical significance was considered at *p* value < 0.05.

## 3. Results

### 3.1. Cystine and Methionine Deficiency Promotes Ferroptosis in Hepatocyte-Derived Cells

**Figure 1:**
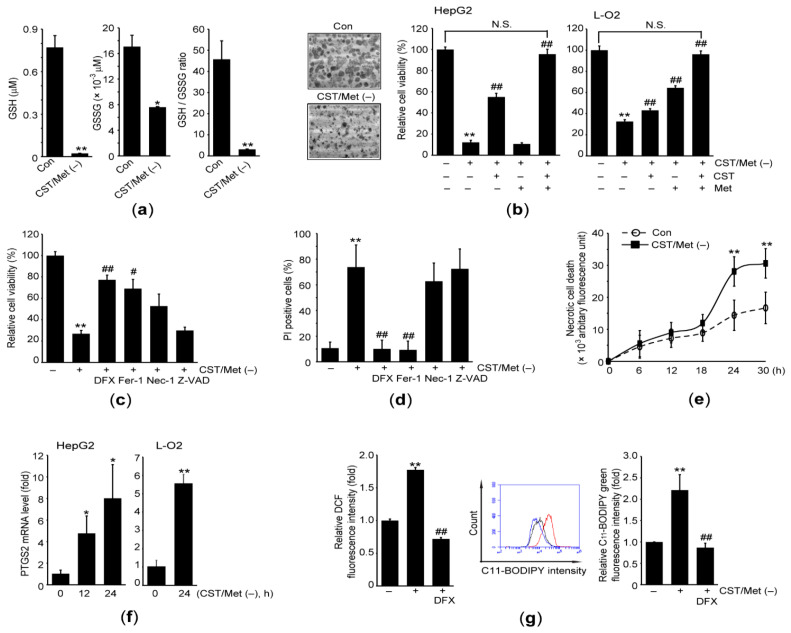
Cystine and methionine deficiency induces ferroptosis of hepatocyte-derived cells. HepG2 and L-O2 cells were exposed to cystine (CST) and methionine (Met) deficiency (CST/Met (−)) for 1 (**g**—**left**), 12–24 (**f**—**left**), 24 (**a**,**f**—**right**, and **g**—**middle** and **right**), 0–30 (**e**), or 36 h (**b**–**d**). (**a**) Intracellular GSH and GSSG levels. (**b**) Morphological changes were observed under a light microscope after exposing HepG2 cells to CST/Met (−) (left). Effects of CST (200 μM) and Met (200 μM) supplementation on the viability of HepG2 (middle) and L-O2 cells (right) determined by MTT assay. (**c**) Viability of HepG2 cells exposed to CST/Met (−) in the presence of DFX (200 μM), Fer-1 (10 μM), Nec-1 (10 μM), and Z-VAD (20 μM). (**d**) Percentage of HepG2 cells showing high PI staining intensity was revealed by flow cytometer. (**e**) Necrotic cell death. Open circle and closed square indicate HepG2 cells incubated with control medium and medium without CST/Met, respectively. (**f**) Level of PTGS2 mRNA in both HepG2 (left) and L-O2 cells (right) determined by qPCR. (**g**) ROS (left) and lipid peroxidation (middle and right) were measured using DCFH-DA and C_11_-BODIPY, respectively. Black, red, and blue lines indicate fluorescence intensities from HepG2 cells incubated with control, CST/Met (−), and CST/Met (−) + DFX, respectively (middle). ** *p* < 0.01, * *p* < 0.05, versus control; ## *p* < 0.01, # *p* < 0.05, versus CST/Met (−): Con, control; DFX, deferoxamine mesylate; Fer-1, ferrostatin-1; Nec-1, necrostatin-1; N.S., not significant; Z-VAD, Z-VAD-FMK.

Prior to investigating the cellular effects of CST/Met (−), we measured the intracellular level of glutathione, a representative antioxidant that has a Cys residue. The GSH and GSSG levels, and GSH/GSSG ratio were significantly decreased when HepG2 cells were exposed to medium lacking CST and Met for 24 h (Figure 1a). Up to 24 h, there were no obvious changes in the morphology of HepG2 cells (data not shown). However few HepG2 cells remained in the culture plate after exposure to CST/Met (−) for 36 h (Figure 1b—left). Similarly, MTT assay showed that CST/Met (−) (36 h) significantly reduced the viability of HepG2 cells. Supplementation of CST partly, but significantly, prevented the reduction of cell viability by CST/Met (−), whereas Met supplementation had no effect. Moreover, there was no difference in the viability of HepG2 cells exposed to control medium and CST/Met (−) medium supplemented with both CST and Met (Figure 1b—middle). The preventive effect of CST and Met supplementation against the cell viability reduction caused by CST/Met (−) was confirmed in hepatocyte-derived L-O2 cells (Figure 1b—right).

To clarify what types of cell death are associated with the reduction of cell viability caused by CST/Met (−), HepG2 cells were exposed to CST/Met (−) in the presence of chemical inhibitors of various types of programmed cell death. The CST/Met (−)-mediated decrease in viability of HepG2 cells was significantly blocked by simultaneous treatment with DFX and Fer-1, but not Nec-1 and Z-VAD (Figure 1c). Therefore, ferroptosis may contribute to a reduction in cell viability in response to CST/Met (−). In addition, CST/Met (−) increased the percentage of cells showing high PI fluorescence intensity (e.g., PI positive cells) as well as necrotic impairment (Figure 1d,e), implying that CST/Met (−) disrupts plasma membrane permeability. However, the CST/Met (−)-mediated increase in PI positive cells was significantly attenuated by treatment with DFX and Fer-1 (Figure 1d). To confirm the association of ferroptosis with CST/Met (−)-mediated cell death, we investigated the effect of CST/Met (−) on major phenotypic markers of ferroptosis. CST/Met (−) significantly increased the mRNA level of prostaglandin-endoperoxide synthase 2 (PTGS2) in HepG2 and L-O2 cells (Figure 1f). In addition, CST/Met (−)-induced ROS production and lipid peroxidation were abolished by DFX (Figure 1g).

### 3.2. Cystine and Methionine Deficiency Activates ISR in Hepatocyte-Derived Cells

**Figure 2:**
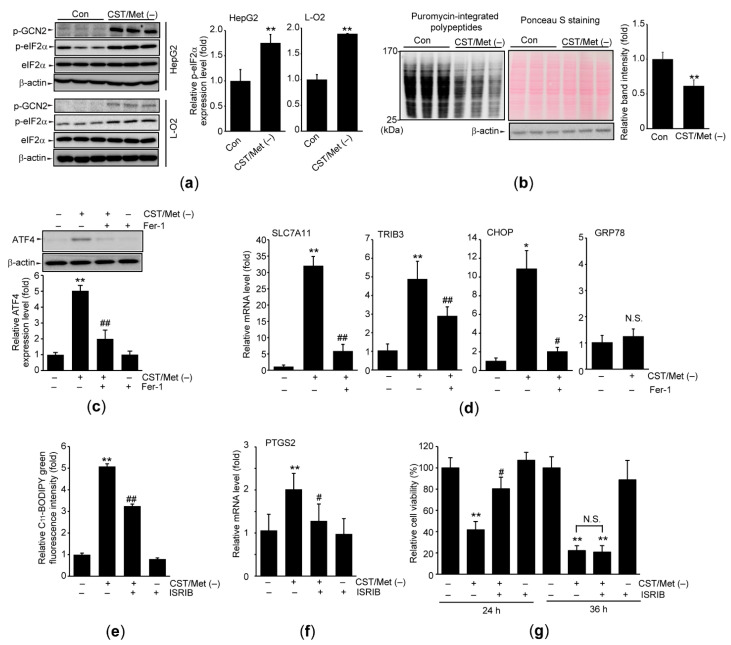
Cystine and methionine deficiency activates the integrated stress response (ISR). HepG2 and L-O2 cells were exposed to CST/Met (−) for 6 (**a**,**b**), 12 (**d**,**f**), 24 (**c**,**e**), or 24–36 h (**g**). (**a**) Phosphorylation of GCN2 and eIF2α. Equal protein loading was verified by β-actin immunoblotting. (**b**) SUnSET assay. Puromycin-integrated polypeptides of 25–170 kDa were quantified by densitometry (left and right). Equal protein loading was verified by Ponceau S staining (upper middle) and β-actin immunoblotting (lower middle). (**c**) ATF4 expression in cells exposed to CST/Met (−) with or without Fer-1 (10 μM) was normalized to β-actin. (**d**) mRNA levels of ISR target genes were determined by qPCR analysis. (**e**–**g**) Lipid peroxidation (**e**), PTGS2 mRNA (**f**), and cell viability (**g**) were determined after HepG2 cells were exposed to ISRIB (1 μM) under CST/Met (−). ** *p* < 0.01, * *p* < 0.05, versus control; ## *p* < 0.01, # *p* < 0.05, versus CST/Met (−): Con, control; ISRIB, ISR inhibitor; N.S., not significant.

GCN2-eIF2α-ATF4 is the canonical signaling axis of ISR induced by amino acid deprivation [17]. CST/Met (−) significantly increased the phosphorylation of GCN2, as well as its downstream substrate, eIF2α, in HepG2 cells. CST/Met (−)-dependent eIF2α phosphorylation was verified in L-O2 cells (Figure 2a). In addition, SUnSET assay showed that CST/Met (−) significantly decreased the expression of puromycin-integrated polypeptides (Figure 2b), indicating that CST/Met (−) halts global protein translation. Moreover, CST/Met (−) upregulated the expression of ATF4, and this upregulation was significantly inhibited by Fer-1 treatment (Figure 2c). CST/Met (−) did not alter the mRNA level of GRP78, a representative target gene primarily regulated by ATF6 under endoplasmic reticulum stress [35], whereas CST/Met (−) increased the mRNA levels of ISR target genes (e.g., solute carrier family 7 member 11 (SLC7A11), tribbles homolog 3 (TRIB3), and C/EBP homologous protein (CHOP)) that are induced by ATF4 [36,37,38]. Furthermore, Fer-1 treatment significantly blocked the induction of ISR target genes (Figure 2d). To investigate the role of ISR signaling pathway in CST/Met (−)-dependent ferroptosis, HepG2 cells were simultaneously treated with ISRIB under CST/Met deficiency. ISRIB significantly decreased lipid peroxidation and PTGS2 induction in response to CST/Met (−) (Figure 2e,f). In addition, CST/Met (−)-mediated reduction of cell viability at 24 h was significantly blocked in the presence of ISRIB. However, the protective effect of ISRIB against cell viability was abolished at 36 h (Figure 2g). Inhibitory effects of ISRIB on ISR were verified by expressions of ATF4 and ISR target genes (Figure S1).

### 3.3. Cystine and Methionine Deficiency Induces BTG1 in an ATF4-Dependent Manner

**Figure 3:**
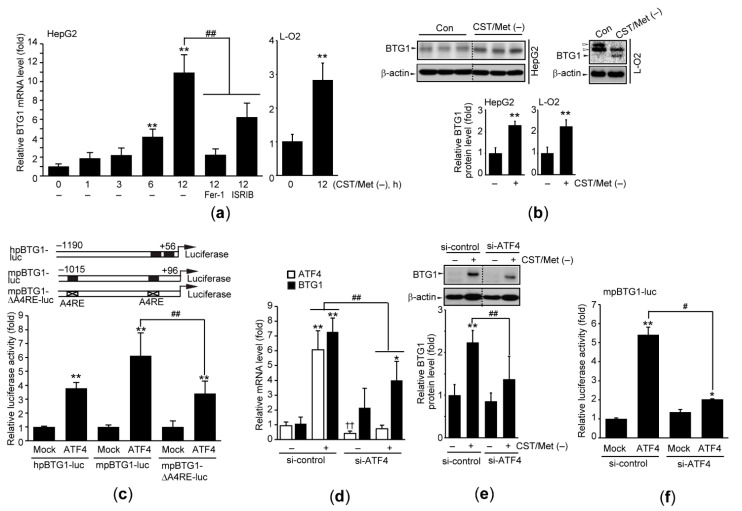
Cystine and methionine deficiency induces BTG1 by activating ATF4. HepG2 and L-O2 cells were exposed to CST/Met (−) for 1–12 (**a**,**d**), or 24 h (**b**,**c**,**e**,**f**). (**a**) The level of BTG1 mRNA was determined by qPCR. Fer-1 (10 μM, 12 h) or ISRIB (1 μM, 12 h) was simultaneously applied to HepG2 cells under CST/Met (−) (left). (**b**) BTG1 protein. Open arrowheads and dashed lines in immunoblot images indicate nonspecific bands and cropped images of the same membrane, respectively (upper). (**c**) BTG1 transactivation. Schematic illustration of constructed reporter plasmids containing the human and murine BTG1 gene promoter (upper). An ATF4 expression plasmid was co-transfected with hpBTG1-luc, mpBTG1-luc, or mpBTG1-ΔA4RE-luc. pCDNA3.2/V5-DEST was used for mock transfection (lower). (**d**–**f**) Effect of siATF4 on BTG1 induction. A siRNA targeting human ATF4 (**d**,**e**) or murine ATF4 (**f**) was transfected into HepG2 cells, and expression of BTG1 (**d**,**e**) and transactivation (**f**) were determined by qPCR, immunoblot, and reporter gene assays, respectively. ** *p* < 0.01, * *p* < 0.05, versus control (**a**,**b**,**d**,**e**) or mock transfection (**c**,**f**); ## *p* < 0.01, # *p* < 0.05, versus HepG2 cells exposed to CST/Met (−) (**a**,**d**,**e**) or ATF4-transfected cells (**c**,**f**); †† *p* < 0.01, between basal level of ATF4 mRNA (**d**): A4RE, putative ATF4 response element; Con, control.

BTG1 is the differentially expressed gene in the liver of rats fed a low-protein diet, and Cys has been reported as an essential amino acid responsible for BTG1 induction [1]. As expected, qPCR analysis showed that CST/Met (−) significantly increased the mRNA levels of BTG1 in HepG2 and L-O2 cells (Figure 3a). In addition, Fer-1 and ISRIB abolished the induction of BTG1 mRNA by CST/Met (−) (Figure 3a—left). Moreover, CST/Met (−) increased the BTG1 protein level in both cell lines (Figure 3b).

To investigate the signaling molecule responsible for CST/Met (−)-mediated BTG1 induction, the human and mouse BTG1 promoter regions were cloned into a reporter plasmid to generate hpBTG1-luc and mpBTG1-luc, respectively (Figure 3c—upper). In addition, in silico promoter analysis using JASPAR2020 was conducted to predict essential transcription factors for BTG1 induction, and we found two putative ATF4 response elements from −1013 to −1000 and −198 to −185 bp of the mouse BTG1 promoter, respectively. Moreover, human and rat BTG1 promoters also contained a putative ATF4 response element (Table 2). Reporter gene assays using hpBTG1-luc and mpBTG1-luc showed that ectopic expression of ATF4 transactivated BTG1 promoter. However, ATF4-mediated transactivation was significantly reduced when HepG2 cells were transfected with the mpBTG1-ΔA4RE-luc, which deleted the two putative ATF4 response elements from the BTG1 promoter (Figure 3c—lower). Furthermore, knockdown of ATF4 using an siRNA significantly decreased CST/Met (−)-induced BTG1 expression (Figure 3d,e), as well as ATF4-mediated transactivation of BTG1 (Figure 3f). Gene silencing by si-ATF4 transfection was verified by observing that si-ATF4 significantly decreased basal and inducible level of ATF4 mRNA (Figure 3d).

### 3.4. BTG1 KO Prevents Ferroptosis Induced by Cystine and Methionine Deficiency

**Figure 4:**
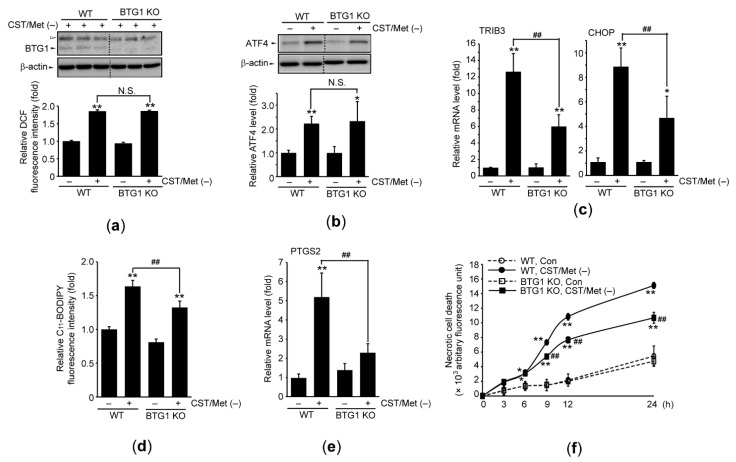
BTG1 induces ferroptosis under cystine and methionine deficiency. WT and BTG1 KO HAP1 cells were exposed to CST/Met (−) for 1 (**a**—lower), 12 (**c**,**e**), 24 (**a**—upper, and **b**,**d**), or 0–24 h (**f**). (**a**) Fluorescence intensity was measured after incubating HAP1 cells with CST/Met (−) and DCFH-DA (lower). The phenotype of BTG1 KO HAP1 cells was verified by BTG1 immunoblotting. Open arrowheads and dashed lines in immunoblot images indicate nonspecific bands and cropped images of the same membrane, respectively (upper). (**b**) Effect of BTG1 KO on CST/Met (−)-inducible expression of ATF4. (**c**) mRNA levels of ISR target genes in HAP1 cells. (**d**–**f**) Lipid peroxidation (**d**), PTGS2 mRNA level (**e**), and necrotic cell death (**f**) in HAP1 cells exposed to CST/Met (−). ** *p* < 0.01, * *p* < 0.05, versus control; ## *p* < 0.01, between HAP1 cells exposed to CST/Met (−); N.S., not significant.

We generated BTG1 KO cells using the CRISPR/Cas9 system, and the phenotype was confirmed by BTG1 immunoblotting (Figure 4a—upper). In addition, Fer-1 decreased CST/Met (−)-mediated necrotic death of WT HAP1 cells, suggesting that ferroptosis is a major player for executing the death of HAP1 cells (Figure S2). When WT and BTG1 KO HAP1 cells were incubated with CST/Met (−) medium and DCFH-DA, fluorescence intensity was increased in both cell lines. There was no significant difference in ROS accumulation between the two cell lines (Figure 4a—lower). Next, we investigated the role of BTG1 in CST/Met (−)-mediated ISR and ferroptosis. Immunoblot analysis showed that the magnitude of the CST/Met (−)-mediated induction of ATF4 protein was not different between the two cell lines (Figure 4b). However, the mRNA levels of TRIB3 and CHOP in BTG1 KO cells, as mediated by CST/Met (−), were significantly lower than those in WT cells (Figure 4c). Moreover, BTG1 KO significantly reduced the lipid peroxidation as well as induction of PTGS2 mRNA in response to CST/Met (−) and prevented CST/Met (−)-mediated necrotic cell death (Figure 4d–f).

## 4. Discussion

Because HepG2 cells lack transsulfuration from Met [25], they can be used to explore the roles of sulfur amino acids in cell viability. In HepG2 cells, the CST/Met (−)-mediated reduction of cell viability was partly, but significantly, restored by CST supplementation, supporting the concept that CST (/cysteine) is more important for maintaining cell viability, whereas both amino acids are required for cell survival. In contrast, in L-O2 cells, the inhibitory effect of Met supplementation on the CST/Met (−)-mediated reduction of cell viability was more potent than that of CST supplementation. This discrepancy is likely a result of compensation for Cys by Met metabolism.

To adapt to CST deficiency, glutathione is rapidly exported from the cell and broken down into Cys to maintain cellular homeostasis [11]. In addition, GSH and GSSG were depleted when HepG2 cells were exposed to CST/Met (−). Moreover, although we did not measure the intracellular iron level, use of DFX (an iron chelator) indicated that iron is associated with CST/Met (−)-mediated lipid peroxidation, ROS production, and cell death. In addition to DFX, Fer-1 also inhibited the decrease in cell viability caused by CST/Met (−) in HepG2 cells. Furthermore, the mRNA level of PTGS2 was increased in HepG2 and L-O2 cells exposed to CST/Met (−). Because glutathione depletion, PTGS2 induction, and lipid peroxidation are all phenotypic changes accompanying ferroptosis [5,10,22,39], our results imply that CST/Met (−) induces ferroptosis in hepatocyte-derived cells.

Ferroptosis shares common features with other regulated death signaling pathways, such as uptake of impermeable fluorescent dyes (e.g., PI) and release of lactate dehydrogenase [5,11,22,40]. Similarly, the percentage of PI positive cells and necrotic cell death were increased by CST/Met (−). In addition, the increase in PI positive cells induced by CST/Met (−) was decreased by Fer-1. Therefore, permeabilization (or leakage) of plasma membrane by CST/Met (−) is dependent on ferroptosis. More importantly, deficiency of amino acids, including CST, leads to activation of the ISR, halts global protein translation, and transactivates ATF4-dependent target genes [11,17]. Because SLC7A11 comprises 12 membrane-spanning regions of a system X_c_^-^ antiporter that take up CST for glutathione biogenesis, ATF4-dependent SLC7A11 induction under CST deficiency is regarded as an adaptive response [5,11,37]. In addition, CHOP and TRIB3 are other target genes primarily regulated by ATF4 [36,38]. We showed that CST/Met (−) phosphorylated GCN2 and eIF2α, downregulated global protein translation, and increased the expression of ATF4 as well as its target genes. Moreover, Fer-1 blocked the induction of ATF4, SLC7A11, CHOP, and TRIB3 by CST/Met (−), implying that the CST/Met (−)-mediated ferroptotic signaling pathway is involved in ISR activation. Furthermore, to explore the casual relationship between ISR and ferroptosis, we used ISRIB and found that ISRIB was capable to inhibit CST/Met (−)-mediated lipid peroxidation and PTGS2 induction. In parallel with a previous report that ISRIB modulates ISR within a defined window of activation [41], present results also showed that ISRIB only delayed the decrease in cell viability by CST/Met (−). Therefore, these results provide evidence that ISR is a functional downstream process for executing ferroptosis in response to CST/Met (−).

In parallel with a previous report [1], we showed that CST/Met (−) significantly induced BTG1 in HepG2 and L-O2 cells. In addition, our preliminary study indicated that sorafenib and erastin, inducers of ferroptosis [10,12,16,40], can increase BTG1 in HepG2 cells (data not shown). Moreover, our results show that Fer-1 prevented CST/Met (−)-mediated BTG1 induction, which suggests that BTG1 can serve as a novel marker of ferroptosis.

BTG1 expression is known to be transcriptionally regulated by FoxO3a and CREB transcription factors [26,34]. In this study, ectopic expression of ATF4 transactivated BTG1 promoter-driven luciferase, while ISRIB treatment or silencing of ATF4 blocked CST/Met (−)-mediated BTG1 expression, implying that ATF4 is essential for BTG1 induction in response to CST/Met (−). In addition, in silico analysis using JASPAR2020 [42] indicated that ATF4 response elements were present within –1.2 kb of the human, mouse, and rat BTG1 promoter, in relatively high scores, suggesting that ATF4 is an evolutionally conserved transcription factor that regulates BTG1. Moreover, BTG1 transactivation by ATF4 was significantly decreased when the two putative ATF4 response elements were truncated in the BTG1 promoter. However, because ATF4-dependent inducibility remained in mpBTG1-ΔA4RE-luc to some extent, we cannot exclude the possibility that ATF4 facilitates BTG1 transcription by directly binding to other unidentified elements in the promoter, or by activating other ATF4-dependent transcription factors.

Although accumulating evidence suggests that BTG1 interacts with ATF4 [25,26], the role of BTG1 in the regulation of ATF4 is controversial. For instance, adenoviral delivery of the BTG1 gene in liver tissue reduces basal expression of ATF4-dependent target genes [26]. By contrary, BTG1 induction by depletion of glutamine enhances the DNA binding ability of ATF4, which is associated with methylation of ATF4 at Arg^239^ via BTG1-dependent recruitment of PRMT1 [25]. In agreement with prior reports [25,26], we showed that BTG1 deficiency did not alter the ATF4 expression induced by CST/Met (−). Moreover, CST/Met (−)-mediated induction of CHOP and TRIB3 mRNA was reduced in BTG1 KO cells, supporting the concept that BTG1 acts as a reciprocal feed-forward activator of ATF4. Further studies are needed on the adaptor molecules (e.g., PRMT1) linking BTG1 and ATF4 in CST/Met (−), because BTG1 does not possess catalytic domains for modifying ATF4 activity.

Beyond adaptation, ISR upregulates cellular death signaling in a manner involving CHOP and TRIB3 genes. CHOP and CHOP/ATF4 heterodimers transactivate the expression of proapoptotic proteins, including PUMA, Bim, death receptor 5, and ATF5 [17,43,44,45,46]. In addition, TRIB3 sensitizes cell to tumor necrosis factor-mediated cell death and inhibits Akt and nuclear factor-κB phosphorylation [47,48]. Although the major form of programmed cell death triggered by CHOP and TRIB3 is apoptosis, upregulation of CHOP and TRIB3 occurs during ferroptosis [5,11]. We showed that BTG1 deficiency suppressed CST/Met (−)-mediated lipid peroxidation, PTGS2 induction, and necrotic cell death. Although the role of BTG1 in ferroptotic death requires further investigation in other experimental systems (e.g., BTG1 overexpressed cells), current results suggest that CST/Met (−)-mediated BTG1 induction likely accelerates ferroptosis by upregulating CHOP and TRIB3.

## 5. Conclusions

In conclusion, the present study showed that CST/Met (−) induced ferroptotic death of hepatocytes via GSH depletion, lipid peroxidation, and PTGS2 induction. In addition, the ISR signaling pathway contributed to facilitating CST/Met (−)-mediated ferroptosis. Finally, we proved that ATF4-dependent BTG1 induction in response to CST/Met (−) promoted ferroptosis of hepatocytes. Therefore, regulation of BTG1 expression may constitute an alternative molecular approach for managing ferroptosis-mediated liver injury.

## Figures and Tables

**Table 1:** Oligonucleotide sequences used in the present study.

**qPCR Analysis**				
**Gene Symbol**	**Forward Primer**	**Backward Primer**	**Accession** **Number**	**Product Size (bp)**
ATF4	5′-GTTCTCCAGCGACAAGGCTA-3′	5′-TCTCCAACATCCAATCTGTCC-3′	NM_001675.4	119
BTG1	5′-AGCGGATTGGACTGAGCAG-3′	5′-GGTGCTGTTTTGAGTGCTAC-3′	NM_001731.2	161
CHOP	5′-GCACCTCCCAGAGCCCTCACTCTCC-3′	5′-GTCTACTCCAAGCCTTCCCCCTGCG-3′	NG_027674.1	422
GAPDH	5′-GAAGGTGAAGGTCGGAGTC-3′	5′-GAAGATGGTGATGGGATTTC-3′	NM_002046.4	226
GRP78	5′-TGCTTGATGTATGTCCCCTTA-3′	5′-CCTTGTCTTCAGCTGTCACT-3′	NM_005347.5	303
PTGS2	5′-ATATGTTCTCCTGCCTACTGGA-3′	5′-GCCCTTCACGTTATTGCAGAT-3′	NM_000963.3	108
SLC7A11	5′-TGCTGGGCTGATTTATCTTCG-3′	5′-GAAAGGGCAACCATGAAGAGG-3′	NM_014331.4	114
TRIB3	5′-TGTCTTCGCTGACCGTGAGA-3′	5′-ACGCGTGCTTGTCCCACAGG-3′	NM_021158.5	101
**Construction of Recombinant Plasmids**				
**Plasmid** **Name**	**Forward Primer**	**Backward Primer**		
hpBTG1-luc	5′-CTCGAGACCCTGTCTTAGGCCTAATCG-3′	5′-AGATCTTCCAGCTCCGCAGCATTCGAA-3′		
mpBTG1-luc	5′-CTCGAGGTGGTGTGTATTGCATCTGATGACC-3′	5′-AGATCTCACATCGCTCGGACCTCCCCAGCC-3′		
* mpBTG1- ΔA4RE-luc	5′-GCCTCGAGGTGGTGTGTGATGACCAACTAAACTC-3′ 5′-GGATGAGAGGGAGGTGCTTGTAAACAAATAAACCCCC-3′	5′-GAGTTTAGTTGGTCATCACACACCACCTCGAGGC-3′ 5′-GGGGGTTTATTTGTTTACAAGCACCTCCCTCTCATCC-3′		

The underlined text indicates recognition sequence for restriction enzymes. * Two sets of primer pairs were used to delete two putative ATF4 response elements in the mouse BTG1 promoter of mpBTG1-luc: GAPDH, glyceraldehyde-3-phosphate dehydrogenase; GRP78, glucose regulatory protein 78.

**Table 2:** Putative ATF4 response elements in BTG1 promoter.

Species	Chromosomal Location	Matrix ID	Score	Relative Score	* Binding Site (bp)	** Orientation	DNA Sequence
*Homo* *sapiens*	chr12:92,145,847–92,147,046	MA0833.1	13.566	0.9104	−197 to −185	–	5′-AGCTGACGTAATC-3′
		MA0833.2	10.3809	0.8701	−198 to −185	–	5′-AGCTGACGTAATCC-3′
		MA0833.1	11.1549	0.8767	−115 to −103	–	5′-TTGTGACGCTTGC-3′
*Mus* *musculus*	chr10:96,615,806–96,617,005	MA0833.2	10.6486	0.8737	−1013 to −1000	–	5′-ATCAGATGCAATAC-3′
		MA0833.1	9.3852	0.8519	−1012 to −1000	–	5′-ATCAGATGCAATA-3′
		MA0833.1	13.566	0.9104	−197 to −185	–	5′-AGCTGACGTAATC-3′
		MA0833.2	11.1591	0.8806	−198 to −185	–	5′-AGCTGACGTAATCT-3′
*Rattus norvegicus*	chr7:37,811,631–37,812,830	MA0833.1	13.566	0.9104	−516 to −504	–	5′-AGCTGACGTAATC-3′
		MA0833.2	11.1591	0.8806	−517 to −504	–	5′-AGCTGACGTAATCT-3′

* −1 indicates the first upstream nucleotide of the transcriptional start site. ** Minus sign indicates that the putative site locates in reverse and complement orientation.

## Data Availability

The data presented in this study are available on request from corresponding author. The data are not publicly available given that we do not have webpages created for such purpose.

## Supplementary Materials

The following are available online at https://www.mdpi.com/article/10.3390/antiox10101543/s1, Figure S1: Effect of ISRIB on the expression of ATF4 and ISR target genes, Figure S2: Effect of Fer-1 on CST/Met (−)-mediated necrotic death of WT HAP1 cells, Figure S3: Original images for immunoblots.
