# Supplementary material for: Cystine and Methionine Deficiency Promotes Ferroptosis by Inducing B-Cell Translocation Gene 1
Source: Antioxidants (Basel). 2021 Sep 28;10(10):1543. doi: 10.3390/antiox10101543 (PMC8532826; doi:10.3390/antiox10101543)
Supplement: Supplementary file 1 [file antioxidants-10-01543-s001.zip › antioxidants-1398674-supplementary.pdf]

antioxidants

Supplementary Material: Cystine and Methionine Deficiency Promotes Ferroptosis by Inducing B Cell Translocation Gene 1

Il-Je Cho [1,†], Doyeon Kim [1,†], Eun-Ok Kim [1], Kyung-Hwan Jegal [1,2], Jae-Kwang Kim [1,3], Sang-Mi Park [1], Rongjie Zhao [4], Sung-Hwan Ki [5], Sang-Chan Kim [1,*] and Sae-Kwang Ku [1,*]

1 College of Korean Medicine, Daegu Haany University, Gyeongsan 38610, Korea; skek023@dhu.ac.kr (I.-J.C.); dy940716@gmail.com (D.K.); keo84@hanmail.net (E.-O.K.); outshinerz@gmail.com (K.-H.J.); kimjk@kiom.re.kr (J.-K.K.); miya38@nate.com (S.-M.P.)

2 Digital Health Research Division, Korea Institute of Oriental Medicine, Daejeon 34054, Korea

3 Korean Medicine-Application Center, Korea Institute of Oriental Medicine, Daegu 41062, Korea

4 Department of Psychopharmacology, Qiqihar Medical University, Qiqihar 161006, China; Zhao_rongjie@yahoo.com

5 College of Pharmacy, Chosun University, Gwangju 61452, Korea; shki@chosun.ac.kr

* Correspondence: sckim@dhu.ac.kr (S.-C.K.); gucci200@dhu.ac.kr (S.-K.K.); Tel.: +82-53-819-1862 (S.-C.K.); +82-53-819-1549 (S.-K.K.)

† Contributed equally to this work.

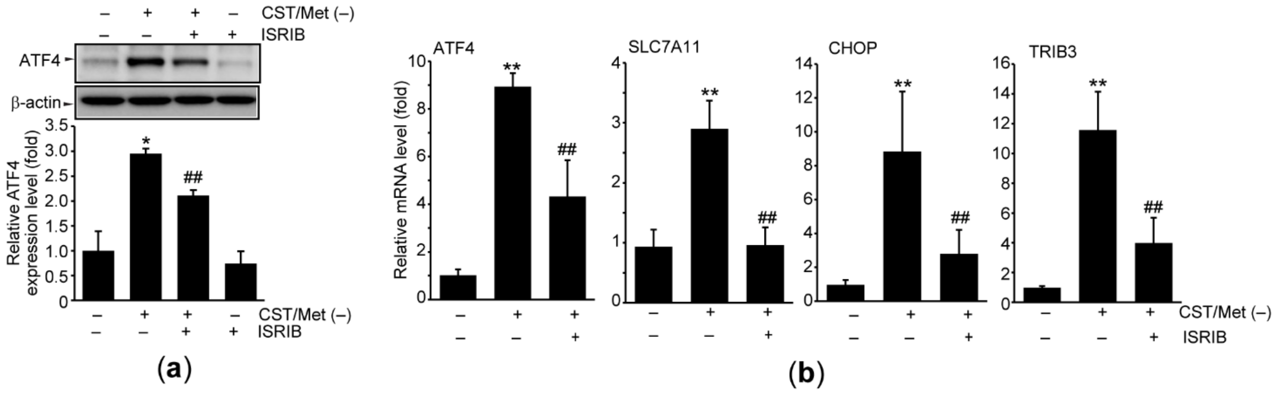

Figure S1. Effect of ISRIB on the expression of ATF4 and ISR target genes. HepG2 cells were exposed to CST/Met (–) for 12 (**b**) or 24 h (**a**) in the presence of ISRIB (1 μM). Protein level of ATF4 (**a**) and mRNA levels of ISR target genes (**b**) were quantified by immunoblot and qPCR analyses, respectively. ** $p < 0.01$, * $p < 0.05$, versus control; ## $p < 0.01$, versus CST/Met (–): ISRIB, ISR inhibitor.

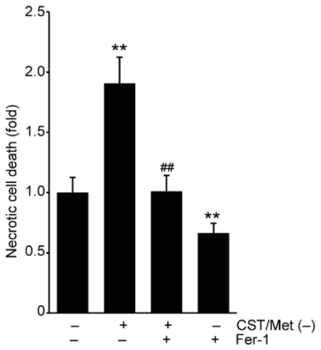

Figure S2. Effect of Fer-1 on CST/Met (–)-mediated necrotic death of WT HAP1 cells. WT HAP1 cells were exposed to CST/Met (–) for 12 h in the presence of Fer-1 (100 μM). Necrotic death was quantified by measuring fluorescence intensity at 485 (emission) and 530 (excitation) nm. ** $p < 0.01$, versus control; ## $p < 0.01$, versus CST/Met (–): Fer-1, ferrostatin-1.

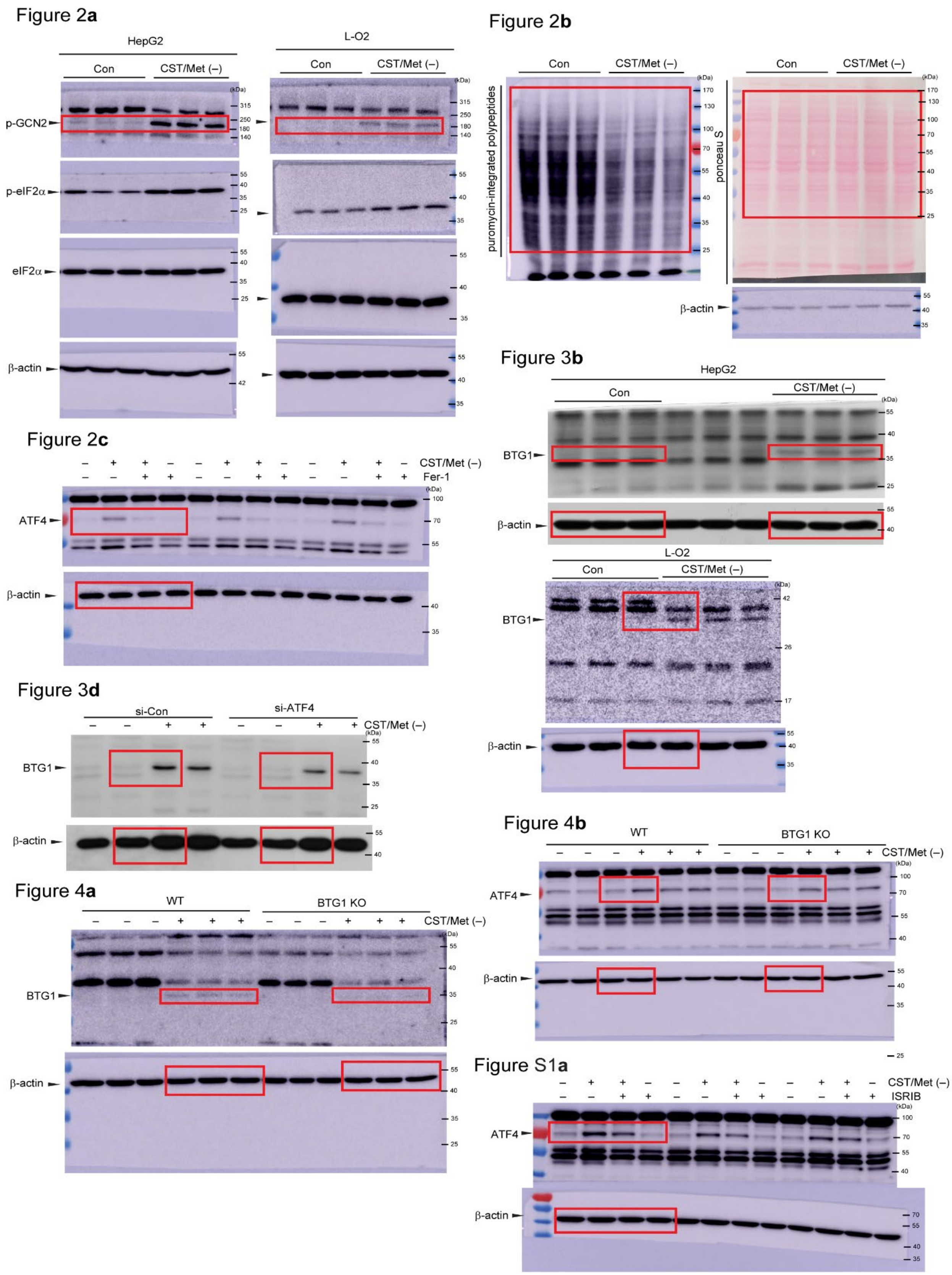

Figure S3. Original images for immunoblots. Red rectangles in original immunoblot images were cropped and used for figures.
